# An investigation of gantry angle data accuracy for cine‐mode EPID images acquired during arc IMRT

**DOI:** 10.1120/jacmp.v15i1.4507

**Published:** 2014-01-06

**Authors:** Peter M. McCowan, Daniel W. Rickey, Pejman Rowshanfarzad, Peter B. Greer, William Ansbacher, Boyd M. McCurdy

**Affiliations:** ^1^ Department of Physics and Astronomy University of Manitoba Winnipeg MB Canada; ^2^ Medical Physics CancerCare Manitoba Winnipeg MB Canada; ^3^ Department of Radiology University of Manitoba Winnipeg MB Canada; ^4^ School of Physics University of Western Australia Crawley Australia; ^5^ Department Radiation Oncology Calvary Mater Newcastle Hospital Newcastle Australia; ^6^ Department of Medical Physics British Columbia Cancer Agency Victoria BC Canada; ^7^ Department of Physics & Astronomy University of Victoria Victoria BC Canada

**Keywords:** gantry angle, linac, EPID, experimental, measurement

## Abstract

EPID images acquired in cine mode during arc therapy have inaccurate gantry angles recorded in their image headers. In this work, methods were developed to assess the accuracy of the gantry potentiometer for linear accelerators. As well, assessments of the accuracy of other, more accessible, sources of gantry angle information (i.e., treatment log files, analysis of EPID image headers) were investigated. The methods used in this study are generally applicable to any linear accelerator unit, and have been demonstrated here with Clinac/Trilogy systems. Gantry angle data were simultaneously acquired using three methods: i) a direct gantry potentiometer measurement, ii) an incremental rotary encoder, and iii) a custom‐made radiographic gantry‐angle phantom which produced unique wire intersections as a function of gantry angle. All methods were compared to gantry angle data from the EPID image header and the linac MLC DynaLog file. The encoder and gantry‐angle phantom were used to validate the accuracy of the linac's potentiometer. The EPID image header gantry angles and the DynaLog file gantry angles were compared to the potentiometer. The encoder and gantry‐angle phantom mean angle differences with the potentiometer were $0.13^{\circ }\pm 0.14^{\circ }$ and $0.10^{\circ }\pm 0.30^{\circ }$, respectively. The EPID image header angles analyzed in this study were within $\pm 1^{\circ }$ of the potentiometer angles only 35% of the time. In some cases, EPID image header gantry angles disagreed by as much as 3° with the potentiometer. A time delay in frame acquisition was determined using the continuous acquisition mode of the EPID. After correcting for this time delay, 75% of the header angles, on average, were within $\pm 1^{\circ }$ of the true gantry angle, compared to an average of only 35% without the correction. Applying a boxcar smoothing filter to the corrected gantry angles further improved the accuracy of the header‐derived gantry angles to within $\pm 1^{\circ }$ for almost all images (99.4%). An angle accuracy of $0.11^{\circ }\pm 0.04^{\circ }$ was determined using a point‐by‐point comparison of the gantry angle data in the MLC DynaLog file and the potentiometer data. These simple correction methods can be easily applied to individual treatment EPID images in order to more accurately define the gantry angle.

PACS numbers: 87.53.Kn, 87.55.T‐, 87.56.bd, 87.59.‐e

## I. INTRODUCTION

Radiation treatment is commonly performed with a linear accelerator (linac) equipped with an electronic portal imaging device (EPID). Although the EPID was originally developed to verify patient position via anatomical imaging using the treatment X‐ray beam, it also has properties attractive for use as a dosimetric verification tool.^1^, ^2^ Its integration with the radiation treatment unit, high spatial resolution, and digital output have made it the subject of many dosimetric studies.^(3)^ These dosimetric properties, along with its acquisition speed, have also made the EPID a promising tool for 2D and 3D dose verifications of intensity‐modulated radiation therapy (IMRT) treatments.^1^, ^4^ This would be particular useful during RapidArc (Varian Medical Systems, Palo Alto, CA) or VMAT (Elekta AB, Stockholm, Sweden) treatments,® a type of arc IMRT that involves the simultaneous control of dose rate, gantry speed, and aperture shaping while the treatment gantry rotates about the patient (VMAT also includes collimator control). While the EPID has been shown to function as a good dosimeter, it has primarily been studied for dosimetric applications when acquiring images in integration mode, which produces a single image consisting of a summation of all frames captured during an irradiation. This is appropriate for static‐beam IMRT delivery. However, in order to utilize the EPID as a dosimetric tool that captures the time‐dependent nature of arc IMRT treatments, it must acquire images in cine mode (also known as continuous mode), where sequential images are produced from averaged frames acquired at a user‐defined frequency. Several studies have described the use of cine mode EPID images for the purpose of *in vivo* dosimetry.^1^, ^6^, ^7^, ^8^

Analysis of EPID images as a function of the gantry angle (or time) is necessary in order to reconstruct the three‐dimensional *in vivo* dose delivered to the patient. However, the actual gantry angle of the linac as a function of time (or EPID image) is not readily available during an arc IMRT treatment. In some cases, the linac gantry angle indicators can also be incorrect due to inadequate QA^(9)^ or inherent design limitations. Uncertainty in the projection angles limits the achievable accuracy of the patient dose reconstruction.

Varian's Trilogy and Clinac 2100ix linac models use an analogue potentiometer to measure the linac's physical gantry angle, while its newer TrueBeam linacs use a digital encoder. The EPID image header angles of a Clinac 2100ix/Trilogy linac are updated approximately every 1 to 3 s, in random time increments, due to an analogue‐to‐digital conversion and nonreal‐time communication software. TrueBeam systems update the image header information (including gantry angle) every 10 ms. In principle, the linac potentiometer (or encoder for TrueBeam) is the most accurate reference to the true mechanical gantry angle, but this signal is not easily accessible during treatment.

For Varian Clinac 2100ix/Trilogy linacs, our group has determined that the gantry angle recorded in the DICOM^(10)^ header of each EPID image can differ by as much as 3° from the linac's gantry potentiometer,^(11)^ while other studies have found similar results.^(4)^ This problem was also observed by Mans et al.^(12)^ for Elekta linacs, where a ball‐bearing phantom was used to determine a 0.4 second systematic time lag in gantry angle information supplied by the software (corresponding to a nearly 3° lag at maximum gantry velocity), although it is not clear in that work if there was any additional random component, nor how reproducible the observation was. In general, the large uncertainty in EPID image header gantry data is unfortunate, since it is the source most readily accessible by clinical users. There is a need for an accurate real‐time estimate of gantry angle for dosimetric applications such as three‐dimensional dose reconstruction^12^, ^13^ and also for EPID‐based pretreatment QA applications. This is illustrated by the recent presentation of a technique which used an experimental approach to directly measure the gantry angle with a customized radiographic phantom during pretreatment delivery.^(14)^ This required modification of the treatment plan to enable imaging of the phantom during the delivery.

The purpose of this investigation is to measure the true gantry angle as a function of treatment time for Clinac 2100ix/Trilogy linacs. (Note that access to TrueBeam linacs is currently unavailable at our center.) In addition, we develop methods one could apply to the gantry angles recorded in the header of EPID images, acquired in clinical mode, to correct them to within the clinically accepted tolerance criteria of $\pm 1^{\circ }$.^15^, ^16^ While applied to Varian linacs here, the methods described in this study can be applied to any linac equipped with an EPID.

## II. MATERIALS AND METHODS

### A. Experimental setup

**Figure 1:**
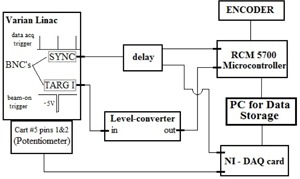
Schematic illustrating how the encoder and potentiometer data were acquired with respect to the clock signal, “SYNC”, and the target current signal, “TARG I”. To convert the target current signal to logic levels and stretch it to a useful length, a level converter was used. This signal allowed us to know when the beam was on or off.

**Figure 2:**
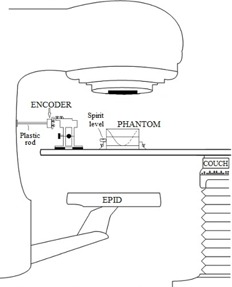
The experimental setup at the linac. An encoder was connected to the center of rotation of the gantry with a plastic rod. The encoder mount allowed for precise lateral and longitudinal adjustments. Target crosshairs machined into the sides and top of our radiographic gantry‐angle phantom allowed positioning at isocenter via room lasers. A spirit level attached to the phantom base, along with adjustable screws located on each of the four support legs, enabled levelling.

**Figure 3:**
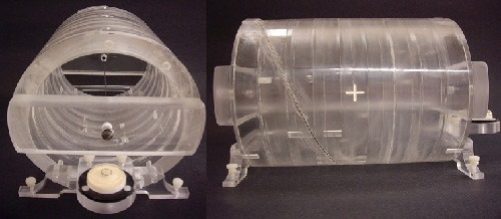
An axial (left) and lateral (right) view of our in‐house constructed radiographic gantry‐phantom. The axial and helical wires are visible, the latter of which was set into grooves precisely machined into the outer frame of the phantom. White levelling screws, a spirit level, and isocenter laser alignment crosshairs are also visible. The hollow cylinder and base were made of acrylic.

**Figure 4:**
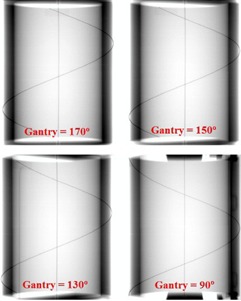
Four inverted gray scale EPID images of the gantry‐angle phantom acquired at gantry angles 170°, 150°, 130°, and 90° (in the International Electrotechnical Commission (IEC) standard). These images illustrate how the intersection points uniquely depend on the gantry angle. The axial (vertical line) and helical (casting a sinusoidal shadow when imaged) tungsten wires are observed.

To fully characterize each linac, we measured the gantry angle data simultaneously with three independent methods that were synchronized with each other. For this work, we used three different Varian Clinac 2100ix linacs (these systems form the basis of Trilogy linacs, so our results are valid for the newer Clinac and Trilogy models) all equipped with EPID's (model aS1000) attached via a mechanical support arm (Exact E‐arm). An overview of the acquisition is shown schematically in Fig. 1 and 2. Each linac provided a convenient clock signal, “SYNC”, which was used to synchronize the rate at which measurements were acquired. Because this signal ran continuously with a frequency determined by the selected dose rate, the target current, “TARG I”, signal was also acquired. The TARG I signal represents the beam pulses produced by the linac. This allowed us to synchronize all acquired data to the same initial point ('beam‐on'). Gantry angle information was then obtained by the following three methods:

The gantry angle was determined from EPID images of an in‐house constructed gantry‐angle phantom (Fig. 3). It consisted of a 0.80 mm diameter tungsten wire wrapped helically around a hollow acrylic cylinder with an additional straight tungsten wire traversing the central axis of the cylinder. The cylinder had a 16.0 cm outer diameter, an inner diameter of 12.8 cm, and a length of 20.0 cm. The phantom was positioned at isocenter by aligning crosshairs etched in the top and sides of its cylindrical frame with the room lasers. A spirit level attached to the base allowed for leveling via adjustable screws in each leg. EPID images of the phantom yielded unique intersection points of the two wires as a function of gantry angle, as illustrated in Fig. 4. The wire intersection points were determined offline using an algorithm programmed in MATLAB (MathWorks Inc., Natick, MA).
2. We measured the potentiometer voltage that provided the gantry angle signal to the treatment unit. This was done by measuring the corresponding analogue signal, directly from the appropriate circuit board at the console area. The target current signal (“TARG I”) was also simultaneously recorded. These data were acquired to a precision of 16 bits at a rate of SYNC/2 using an analogue‐to‐digital converter board (PCI‐MI0‐16XE‐50 and LabView, National Instruments, Austin, TX). Acquisition commenced before beam‐on and ended after beam‐off. A gantry angle‐to‐voltage conversion factor was calculated by averaging the first 20 data points and last 20 data points, which resulted in a range of 17.795±0.007V for a 359.8° rotation.
3. The mechanical gantry angle was also physically measured using an optical incremental rotary encoder (SM23‐2500‐50/5, DATA TECH, Billerica, MA) adhered to the center of rotation of the linac gantry, as illustrated in Fig. 2. The encoder had a resolution of 10,000±1 counts per revolution and data were acquired at a rate of SYNC/6 using a microcontroller (MiniCore RCM5700, Digi International, Minnetonka, MN) outfitted with an additional 2 MB of storage. The microcontroller also acquired the target current signal (“TARG I”), thus enabling encoder measurements to be synchronized to the measurements of potentiometer voltage.

The above three measurement‐based methods were then compared to the angles recorded in the treatment console multileaf collimator (MLC) DynaLog file and the angles in the EPID image headers, both of which were created during treatment delivery. The MLC DynaLog file from a Clinac 2100ix/Trilogy linac records the gantry angle and MLC positions every 50 ms,^(17)^ while the EPID image acquisition details are discussed in Materials and Methods section C.1 below. Our goal was to determine any errors these data exhibited with respect to the true gantry angle.

### B. Gantry‐angle phantom

#### B.1 Intersection detection algorithm

**Figure 5:**
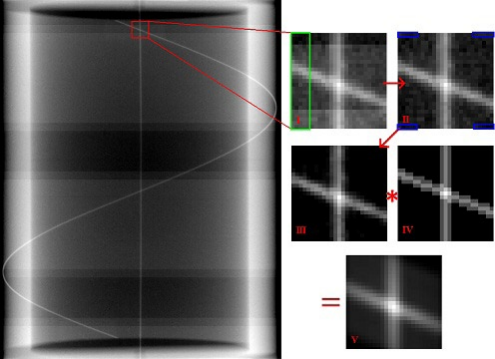
An illustration of the wire intersection detection algorithm. The original image, which may contain banding artifacts, was cropped to a 25×25 pixel region of interest (I). Banding artifacts were removed by looking for adjacent row average differences, located within the green rectangle of image I, larger than a given threshold value. A background noise reduction was applied to the ROI by subtracting the maximum pixel value of the blue boxes from image II. A 2D convolution of image III with a reference image (IV) was calculated to produce a final image (V). A centroid calculation was performed on a 5×5 pixel square centered on the maximum intensity pixel in image V to determine the intersection coordinate.

Figure 4 illustrates the movement of the wire intersection points for several projection images of the gantry‐angle phantom. Only the wire intersection at the top of the Fig. 4 image was evaluated since, for a full arc, these points remained in the image field of view the whole time, unlike the other intersection point which exited the bottom or top of the image halfway through an arc. A stepwise procedure of the algorithm used to locate the intersection points is explained below with the aid of Fig. 5. In most cases these intersections were also confirmed manually.

In the main image of Fig. 5, the intersection point was found by creating a 25×25 pixel region of interest (ROI) around its expected location (user input was required for the first point only). The algorithm also required the number of frames averaged and the direction of rotation. From these, boundary conditions for the approximate location of subsequent intersection points were calculated and used for more accurate analysis as described below.

The ROI (I) was centered on the estimated coordinates of the new intersection point determined from the previous step. Unfortunately acquisition of EPID images in cine mode produces banding artifacts, resulting from a lack of synchronization between the irradiation pulses and detector readout.^18^, ^19^, ^20^ These are observed in Fig. 5 and were present, with differing severity, in our images. The ROI was processed to remove any banding artifacts. These were identified in the algorithm by averaging each row's first five pixel values (green box on image I in Fig. 5) and looking for differences between adjacent row averages that were larger than ±15 grayscales, a threshold manually determined by trial and error. When average row differences were larger than the threshold, the average row value was added to the remaining rows of pixels above or below that row. Values for background noise were determined by identifying the maximum pixel value from four rows of five pixel values, located at each corner of the ROI, and subtracted from image II (blue boxes in Fig. 5).

A two dimensional convolution was performed between the processed ROI image (III) and a reference image (IV) representing a perfect projection of the wire intersection at the expected gantry angle. The convolution produced an image (V) where the greatest pixel intensity was located at the wire intersection point.

A 5×5 pixel ROI was then centered on the maximum intensity pixel of the convolution result. A center‐of‐mass calculation for pixel intensity of the 5×5 ROI was performed to give subpixel accuracy in the X and Y directions.

Due to the constant image acquisition frequency, rotation direction constraints were added in order to reduce incorrect detections of the intersection points. It was observed that consecutive EPID images (with three‐frame averaging) resulted in y‐axis intersection points that differed from their previous location by no more than five pixels. Consecutive intersection points along the y‐axis should increase for counterclockwise rotations and decrease for clockwise rotations. Consequently, any point outside these limits was deemed invalid, rejected, and the algorithm cycled using the next maximum intensity pixel of the convolution result until a point within the limits was found.

Each intersection coordinate was converted to a gantry angle using a scale factor determined from the initial and final coordinates of the gantry‐angle phantom's intersections at the start and end angles of the arc.

Due to the effects of gravity, there is a measureable flex of the EPID imager and support arm, which is reproducible and can be quantified as a function of gantry angle.^11^, ^21^ The amplitude of the resulting y‐axis (y‐axis of the EPID is parallel to the gantry's axis of rotation) shift of the EPID was roughly one pixel or about 0.39 mm, and has the form:

(1) $$Y_{\text{corr}}=Y_{\text{meas}}+(0.39\;\text{mm})\cdot \text{cos}(\theta_{g})$$

where $\theta_{g}$ is the gantry angle in IEC units, $Y_{\text{meas}}$ is the y‐axis intersection point determined from our algorithm, and $Y_{\text{corr}}$ is the EPID flex corrected intersection point. We incorporated this correction in our analysis. Since the wire intersection translation was only along the y‐axis, there was no need to perform an x‐axis correction.

#### B.2 Algorithm accuracy

The intersection point detection algorithm was independently verified by acquiring EPID images of the phantom at fixed (i.e., static) gantry angles. The resulting gantry angles calculated by the algorithm were compared to the gantry angles displayed on the treatment console computer and to the gantry angles calculated from manually determined intersection points. The images were acquired at 10° intervals over a 360° arc. In addition, the accuracy of the cardinal angles (0°, 90°, 180°, and 270°) was independently validated with a high precision, four‐sided gantry level (F#352‐200; Radiation Products Design, Albertville, MN) accurate to 1/40 of a degree.

#### B.3 Phantom irradiation

For data acquisition, 6 MV beams delivering 700 MU at 600 MU/min were used to capture continuous (cine) EPID images. To determine the initial and final intersection points for the gantry‐angle phantom, two additional images were acquired with 100 MU at 600 MU/min for fixed gantry angles (179.9° and 180.1°) before and after delivering the treatment arc. The EPID was positioned at 140 cm source‐to‐detector distance (SDD). Frames were acquired at high resolution (1024×768pixels or 0.39 mm/pixel), which was necessary to accurately determine the gantry‐angle phantom's intersection points. The ‘BeamOnDelay’ parameter of Varian's Image Acquisition System v.3 software was set to zero.

A RapidArc plan (Varian Medical Systems) was constructed specifically for this study using the Eclipse software package. It consisted of a 359.8° arc with fixed jaws and MLCs producing a 21.5 cm (y‐axis) by 26.0 cm (x‐axis) field at isocenter (100 cm SAD) in order to image the entire gantry‐angle phantom. Using three linacs, there were a total of 16 trials (arcs) delivered in treatment mode, consisting of seven counterclockwise and nine clockwise rotations.

### C. Time synchronization

To allow comparison of data collected with the different methods, each set of data needed to be referenced to ‘beam‐on'. The target current signal, TARG I, is synchronized directly to the SYNC signal. The SYNC signal is the master timing signal for the linac's electronic control system. For a dose rate of 600 MU/min, the SYNC signal frequency will be 360 Hz (it decreases by 60 Hz for every decrease of 100 MU/min). Thus, the acquisition rates for the potentiometer and encoder data were 180 Hz and 60 Hz, respectively. DynaLog files update linac angle data at 20 Hz (for all dose rates), which was assumed to be derived from the SYNC signal. The linacs create separate DynaLog files for each arc and the file only records angle data during beam‐on. We set the EPID frame acquisition rate to 7.5 Hz, which is also derived from the SYNC signal.^(20)^ EPID images saved by the clinical computer system were three‐frame averaged (i.e., acquired at 2.5 Hz). This was chosen due to a memory limitation of the On‐Board Imager (OBI) computer,^(22)^ which limits the total number of high‐resolution images in a single treatment delivery that can be acquired, to roughly 200. This limitation of cine mode imaging with a Varian Clinac 2100ix/Trilogy was also observed in another study.^(14)^ Consequently, approximately 185 EPID images were produced for each 359.8° arc.

#### C.1 EPID image time uncertainty

Since data were acquired at submultiples of the SYNC signal, there were uncertainties with respect to the first TARG I pulse. The maximum uncertainty associated with each method could be as large as the acquisition period minus one SYNC period. The maximum time uncertainties for the potentiometer, encoder, and DynaLog file were neglected due to their fast acquisition rates. The slower acquisition rate of the EPID images (2.5 Hz) produced the largest time uncertainties. In order to characterize these, a separate computer with frame‐grabbing hardware and associated software package (iTools Capture, Varian Medical Systems) was connected directly to the frame processing board of the treatment console computer. This allowed us to capture EPID frames continuously before and during beam‐on. Using the same RapidArc plan discussed above, we delivered a total of 10 arcs on the same three linacs (five counterclockwise and five clockwise trials). The EPID images saved by the clinical software were compared to the frames captured by the frame grabber. Our goal was to determine an estimated time delay correction, $t_{d}$, to be applied to the clinically saved EPID images.

### D. Correspondence of the encoder, gantry potentiometer, and gantry‐angle phantom readings

In order to characterize the potentiometer, we compared every third angle measurement made with the potentiometer to angle measurements made with the rotary encoder. We also compared the gantry‐angle phantom data to a two‐point interpolated potentiometer angle dataset.

### E. EPID header and DynaLog file angles

The EPID image header angles were compared to a two‐point interpolation of the potentiometer angle dataset with respect to the EPID image time. Header angles were compared to potentiometer angles three different ways: using the unmodified EPID image time, applying our EPID image time delay correction, $t_{d}$, and by applying a boxcar smoothing function to the time delay corrected EPID image headers. The boxcar function was utilized to smooth noise in the angle data, present due to the random 1 to 3 second update lag for the EPID headers. It convolves the header angle data with a box‐shaped pulse of width (2m+1) values, where m is the number of nearest neighbors. The function had the form:

(2) $$\Delta \theta_{s}(i)=\frac{1}{2m+1}\sum_{j=(i-m)}^{\left(i+m\right)}\left[\theta (i)-\theta (j)\right]$$

where $\Delta \theta_{s}(i)$ is the difference from the unsmoothed gantry angle θ(i), and ‘m’ was chosen to be 2 (for three frame averaging this corresponds to a 1.6 second box‐shaped pulse) in order to minimize errors due to the update lag.

The angle data in the DynaLog file (20 Hz) were compared to every ninth potentiometer angle (180 Hz). Mean angle differences and standard deviations were calculated.

### F. Independent inclinometer measurement

A completely independent gantry angle measurement technique was used on a Clinac 2100ix/Trilogy linac located at a different treatment facility. An inclinometer (NG360; Nordic Transducer, Handsund, Denmark) was bolted to a steel frame, which was attached to the linac head's accessory tray slot such that it could not move during gantry rotation. Potentiometer data were acquired using OMB‐DaqView‐XL software (Omega Engineering Inc., Stamford, CT) with a Personal Daq/56 USB analogue‐to‐digital data acquisition module (IOtech, Norton, MA) attached to the linac's gantry potentiometer. Six rotations were completed (without an encoder or gantry‐angle phantom present), while the inclinometer acquired data at 1.0 s intervals, and the inclinometer data were compared to the interpolated potentiometer data. The manufacturer has stated that there is a 0.56 s systematic lag for this inclinometer model.^(23)^ For each trial, 360 MU at 600 MU/min were delivered over 359.8° arcs. Since inclinometers are popular choices for gantry angle QA we wanted to compare the inclinometer accuracy to that of our encoder and gantry‐angle phantom methods.

## III. RESULTS

### A. Phantom intersection detection algorithm accuracy

Verification of the gantry‐angle phantom and analysis algorithm was carried out by comparing the angles calculated from the gantry‐angle phantom image analysis to angles displayed on the treatment console, in 10° intervals using static gantry angles. Using the high precision gantry level we confirmed that the console displayed the cardinal angles to within $\pm 0.08^{\circ }$. A linear regression performed on the algorithm‐determined gantry angles from the EPID images as a function of the angles displayed on the treatment console resulted in a slope of 1.000±0.143. This indicated that the error in the linac's gantry angle display was random and likely larger than $\pm 0.08^{\circ }$ at angles other than the cardinal angles. As well, a linear regression of manually‐determined gantry angles (i.e., intersection points) to those determined automatically by the algorithm resulted in a slope of 1.000±0.016. The strong agreement in the regression analyses confirms that the algorithm was accurately determining intersection points.

The gantry‐angle phantom analysis was susceptible to mechanical effects inherent to the linac's gantry and EPID support arm. These effects, which differ for each linac, may arise from wear and tear of linac components. These effects do not include gravitational effects on the EPID and EPID arm as those were corrected for in our analysis.

### B. EPID image time delay correction

**Figure 6:**
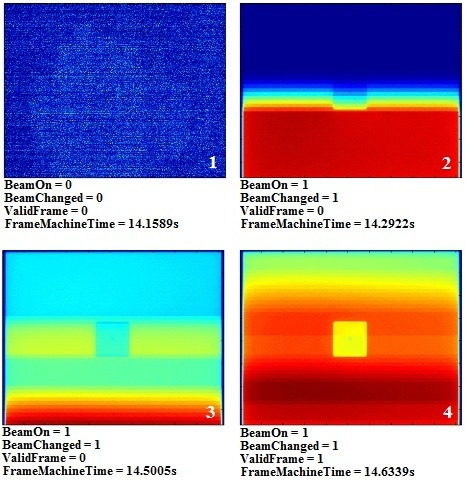
EPID frames and header information of a simple cube phantom acquired at 7.5 Hz using a frame grabber. The jaws and MLCs were positioned to allow full EPID panel irradiation. Frames 2 and 3 were acquired with beam‐on (BeamOn=1) but were not saved to the clinical system (ValidFrame=0). A time delay, $t_{d}$, before the first clinically saved frame (ValidFrame=1), can be calculated from the first two nonvalid beam‐on frames. It is also observed that frame 3 has an acquisition time of 0.2083 s, rather than 0.1333 s.

Comparison of the EPID images saved by the clinical systems with the frame‐averaged images acquired with the frame grabber revealed that up to two frames after beam‐on were tagged ‘invalid’ by the clinical software and thus not saved. Furthermore, the average frame acquisition time of the second invalid beam‐on frame was 0.203±0.004 s for nine of the ten trials, instead of the expected 0.133 s. This is illustrated for one trial in Fig. 6. There was only one case where just a single frame after beam‐on was not saved. This frame had a frame acquisition time of 0.203 s and all the rows had some acquired signal. This was likely due to the beam‐on trigger happening to be in sync with the EPID frame acquisition.

The time when beam‐on occurs for the first unsaved EPID frame is random but can be calculated simply by determining the number of rows of acquired dose and knowing the frame's acquisition time. This is possible because the Varian aS1000 EPID reads pixel data in a row‐by‐row fashion (both Varian and Elekta EPID acquisition details can be found in the work of Podesta et al.^(24)^). We then found that the average time delay before the first clinically saved frame for all ten trials was 0.262±0.042 s.

Since a frame grabber is not available with clinical computer systems, we wanted to estimate this time delay in order to apply it to every day clinical use. Due to the aforementioned frame‐grabber analysis, we estimated $t_{d}$ to be 0.267 s (half of a frame period plus 0.200 s) for two reasons: to minimize the synchronization error between datasets due to random beam‐on times, and because the probability of only a single frame loss after beam‐on was extremely rare. Furthermore, since each clinically acquired EPID image was an average of three frames, the time stamp given to each was the midpoint of the image acquisition period. Thus, the first image was given a time stamp of $T_{1}=t_{d}+0.200\;s=0.467\;s$, and the next image was given a time stamp of $T_{2}=T_{1}+0.400=0.867\;s$. An initial frame delay time of 0.400 s was reported in a study using an Elekta SL20i linac with three frame‐averaged EPID images, but the source of the delay was not determined.^(12)^

### C. Correspondence of the encoder, gantry‐angle phantom, and gantry potentiometer readings

**Figure 7:**
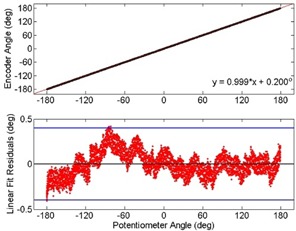
Gantry angles measured with an optical encoder plotted as a function of the gantry angles measured by the linac's potentiometer for one trial. Both acquisition modes agreed on the gantry angle to within $\pm 0.4^{\circ },99.9\%$ of the time (between blue lines). Similar observations were found for all trials. The low frequency variation in residuals is due to slight movements of the gantry covers that could create misalignments between the encoder and the gantry's central axis of rotation. The high frequency residuals are due to electrical noise associated with the potentiometer.

Encoder measurements for one gantry rotation are plotted in Fig. 7 as a function of the corresponding potentiometer measurements. A simple point‐by‐point comparison showed that the encoder measurements agreed with 99.9% of the potentiometer measurements to within $\pm 0.40^{\circ }$. A linear fit resulted in a slope of 0.999±0.018. Also shown are the resulting residuals found by subtracting the potentiometer measurements from the encoder measurements. The blue lines indicate the region in which 99.9% of the measurements lie (i.e., within $\pm 0.40^{\circ }$). Similar results were found for all trials. The low frequency variations in residuals are most likely due to mechanical deformation of the gantry cover creating small offsets between the encoder rod and the gantry's central axis of rotation. The high frequency variation in residuals is due to the electronic noise associated with the analogue potentiometer.

The absolute mean difference and standard deviation in the gantry angle determined by the encoder and the potentiometer for each trial are given in Table 1. One trial for linac #2 had no encoder data acquired. The absolute mean difference in angle and standard deviation between the encoder and potentiometer data for linac #1, #2, and #3 were $0.15^{\circ }\pm 0.12^{\circ }$, $0.07^{\circ }\pm 0.17^{\circ }$, and $0.17^{\circ }\pm 0.12^{\circ }$, respectively.

The absolute mean difference in angle and standard deviation between the gantry‐angle phantom and potentiometer for linac #1, #2, and #3 were $0.10^{\circ }\pm 0.28^{\circ }$, $0.10^{\circ }\pm 0.29^{\circ }$, and $0.09^{\circ }\pm 0.31^{\circ }$, respectively. Results of all trials for all linacs are also given in Table 1. Without incorporating the EPID image time delay correction, $t_{d}$, discussed in Materials and Methods section C.1 above, the absolute mean difference in gantry angle with the potentiometer for linac #1, #2, and #3 were $1.18^{\circ }\pm 0.28^{\circ }$, $1.20^{\circ }\pm 0.29^{\circ }$, and $1.26^{\circ }\pm 0.31^{\circ }$, respectively (not given in Table 1). The significant improvement in angle accuracy validates our time delay correction value.

The high levels of agreement between these two independent measurements with the potentiometer indicate that, as expected, the linac potentiometer is accurately recording the gantry angle and that our potentiometer acquisition method was reliable. For subsequent analyses, the potentiometer was used as the reference for comparison.

**Table 1:** Encoder and gantry‐angle phantom absolute mean differences in angle with the linac potentiometer

		*Encoder*	*Gantry‐Angle Phantom*
*Linac*	*Rotation Direction*	*Absolute Mean Angle Difference* (±SD)	*Absolute Mean Angle Difference* (±SD)
#1	CW1	0.15±0.12	0.01±0.30
	CW2	0.16±0.13	0.12±0.29
	CW3	0.30±0.12	0.10±0.28
	CCW1	0.13±0.12	0.17±0.29
	CCW2	0.01±0.12	0.10±0.26
#2	CW1	0.00±0.17	0.04±0.31
	CW2	n/a	0.07±0.29
	CW3	0.05±0.17	0.07±0.29
	CCW1	0.14±0.17	0.04±0.30
	CCW2	0.04±0.16	0.21±0.28
	CCW3	0.11±0.17	0.17±0.28
#3	CW0	0.24±0.11	0.10±0.30
	CW1	0.15±0.13	0.11±0.31
	CW2	0.30±0.12	0.08±0.30
	CCW1	0.13±0.12	0.05±0.33
	CCW2	0.01±0.12	0.09±0.33

### D. EPID image header angle corrections

The absolute mean difference and standard deviation between the uncorrected $\left(t_{d}=0\right)$ EPID header angles and potentiometer angles for all trials are given in Table 2. Both the mean and standard deviation were given to illustrate the statistical variation of the angle differences. It can be seen that the gantry angles recorded in the EPID image headers have large standard deviations and typically disagree with the potentiometer by more than 1°. For some trials, individual EPID header gantry angles disagreed by as much as $\pm 3^{\circ }$ with the potentiometer, much larger than the tolerance criteria of $\pm 1^{\circ }$.

The percentage of the EPID header gantry angle differences within $\pm 1^{\circ }$ of the potentiometer are also given in Table 2. For linacs #1, #2, and #3, the average percent of uncorrected $\left(t_{d}=0\right)$ EPID header angles within $\pm 1^{\circ }$ of the potentiometer were 38.7%, 36.8%, and 29.3%, respectively, and was as low as 21.6% for one trial. This highlights the need for a method that adjusts the gantry angles recorded in the EPID header to within an acceptable tolerance. Incorporating our suggested time delay correction, the average angle agreement within $\pm 1^{\circ }$ improved to 74.6%, 75.7%, and 75.3% for linacs #1, #2, and #3, respectively. For all linacs, an average of 99.4% of the boxcar smoothed time delay‐corrected EPID header gantry angles agreed within $\pm 1^{\circ }$ of the potentiometer; the lowest agreement was 97.8% for one trial. In all trials, it was either the first and/or last two images which had differences in angle larger than $\pm 1^{\circ }$, but they never exceeded $\pm 1.23^{\circ }$. This was due to the convolution of the boxcar smoothing function not including enough prior or subsequent data points in those regions of the data.

**Table 2:** Differences between the potentiometer and EPID header angles, including time delays and linear fits

		*EPID Image Headers*				
		*Uncorrected* $\left(t_{d}=0\right)$			*Time Delay Corrected* $\left(t_{d}=0.267s\right)$	*Boxcar Time Delay Corrected*
*Linac*	*Trial*	*Absolute Mean Diff.*	*St. Dev.*	$\%\;\text{Within}\pm 1^{\circ }$	$\%\;\text{Within}\pm 1^{\circ }$	$\%\;\text{Within}\pm 1^{\circ }$
#1	CW1	0.95	0.78	49.7	76.1	99.5
	CW2	1.32	0.82	40.2	76.6	99.5
	CW3	1.36	0.81	23.3	75.0	100
	CCW1	1.06	0.89	41.2	73.8	98.9
	CCW2	1.12	0.89	39.0	71.6	97.8
#2	CW1	1.10	0.74	35.6	80.6	100
	CW2	1.01	0.75	46.4	81.8	98.9
	CW3	1.18	0.73	37.0	79.2	100
	CCW1	1.35	0.84	27.9	74.9	98.9
	CCW2	1.06	0.87	43.4	73.6	99.4
	CCW3	1.46	0.90	30.2	64.3	99.4
#3	CW1	1.65	0.78	21.6	75.8	98.9
	CW2	1.18	0.79	40.3	74.3	99.5
	CW3	1.51	0.78	25.8	77.4	100
	CCW1	1.55	0.76	24.6	69.1	98.9
	CCW2	1.24	0.73	34.0	80.1	100

**Figure 8:**
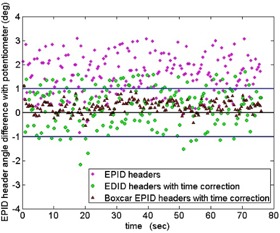
Graph showing differences in angle between the EPID image header data and the linac potentiometer data (black line at zero) as a function of the EPID image time for trial “CW1” from linac #3. Only 21.6% of the unmodified header angles (purple diamonds) were within $\pm 1^{\circ }$ (blue lines) of the potentiometer and had a maximum angle difference of 3.1°. Applying the time correction to the EPID image times (green circles) improved the $\pm 1^{\circ }$ angle agreement to 75.8%, while 98.9% of the boxcar smoothed, time‐corrected header angles (red triangles) agreed within $\pm 1^{\circ }$.

An example of these analyses is illustrated in Fig. 8 for trial “CW1” of linac #3. The EPID image header gantry angle datasets shown include the uncorrected (purple diamonds), the time corrected (green circles), and the time corrected and boxcar smoothed analysis. The blue lines illustrate the defined tolerance of $\pm 1^{\circ }$ agreement with the potentiometer (black line at zero). There is an observed improvement in the header gantry angle accuracy due to the incorporation of our EPID image time delay correction.

### E. DynaLog file's gantry angle accuracy

The absolute mean difference between the DynaLog file gantry angle and the potentiometer gantry angle for linacs #1, #2, and #3 was $0.11^{\circ }\pm 0.04^{\circ }$, $0.12^{\circ }\pm 0.04^{\circ }$, and $0.11^{\circ }\pm 0.04^{\circ }$, respectively. Importantly, 100% of the gantry angle differences were within $\pm 1.0^{\circ }$ of the potentiometer and the largest angle difference was 0.46° for one dataset. Therefore the gantry angle data stored within the MLC DynaLog file was an accurate reference to the true gantry angle. Unfortunately accessing the DynaLog file is far less convenient than accessing the EPID image header information.

### F. Inclinometer results

The angles measured with the inclinometer and the potentiometer had a mean difference of $0.33^{\circ }\pm 0.22^{\circ }$ for all trials. The relatively large standard deviation was due to the measurement method of the inclinometer system, which is based on a liquid capacitive sensor that produces significant electronic noise during movement.

## IV. DISCUSSION

To perform *in vivo* patient dose reconstruction for arc IMRT (RapidArc or VMAT), one needs to accurately know the gantry angle associated with each EPID image. At present, the exact accuracy in EPID image gantry angle required to accurately reconstruct patient dose is unknown. However, we consider the AAPM Task Group 142^(16)^ and Task Group 40^(15)^ recommended gantry angle accuracy of $\pm 1^{\circ }$ to be a logical goal. During irradiation of a patient, it is impractical to have an encoder or gantry‐angle phantom present on the linac couch. This study presents several alternative means of reducing the EPID image gantry angle error. While we utilize the Clinac series linacs in this work, the methods can be applied easily to EPID images acquired with any commercial linac.

The time delay associated with clinically acquired cine mode EPID images using Trilogy/Clinac2100ix linacs was quantitatively measured using a frame grabber. Similar results were found for EPIDs on Elekta linacs.^(12)^ Using our results from the frame‐grabber analysis, we estimated a time delay correction which was used on the EPID images of each trial. We chose this approach, rather than measuring a time delay correction for each trial, in order to make the methods proposed here clinically applicable. Our suggested estimate of the time delay correction to be applied to the time stamp of the EPID image header noticeably increased the percentage of header gantry angle values within $\pm 1^{\circ }$ of the true gantry angle.

We have verified the accuracy of the linac's potentiometer gantry angle during arc IMRT using an optical encoder and with an in‐house constructed gantry‐angle phantom. The difference in angle between the potentiometer and encoder for all linacs was $0.13^{\circ }\pm 0.14^{\circ }$. A difference of $0.10^{\circ }\pm 0.30^{\circ }$ was found between the potentiometer and gantry‐angle phantom for all linacs. The potentiometer accuracy was also verified to within roughly a third of a degree by an inclinometer. Although the encoder and gantry‐angle phantom cannot be used for gantry angle correction during regular treatment, they can alternatively be used for precise linac gantry angle QA.

It has been shown that the gantry angles recorded in the EPID image headers can differ from the potentiometer measurements by as much as $\pm 3^{\circ }$. For the trials presented here, an average of 35% of header angles agreed within $\pm 1^{\circ }$ of the potentiometer. However, by applying our suggested time delay correction to the EPID image time, an average of 75% of the header angles agreed within $\pm 1^{\circ }$ of the potentiometer for all trials. By also applying a simple boxcar smoothing to the time corrected images, an average of 99.4% of the header angles were corrected to within $\pm 1^{\circ }$ of the potentiometer. Alternatively, the gantry angles recorded in the DynaLog file were shown to lie within $\pm 0.5^{\circ }$ of the potentiometer data. Although the DynaLog angle data meet the desired accuracy, it is inconvenient and time‐consuming to retrieve the file for each treatment arc, compared to using the EPID headers.

The inclinometer model used in this study adequately characterized the linac's gantry angle during arc IMRT, although it was less accurate than the encoder or gantry‐angle phantom methods. This was mainly due to its slower acquisition rate and high level of noise.

## V. CONCLUSIONS

We have investigated three independent methods for accurately measuring the linac gantry angle and have developed three approaches to obtain an accurate, on‐treatment EPID image gantry angle for Varian Clinac/Trilogy linacs. We have demonstrated that the gantry angle data contained in both the DynaLog files and in the EPID image headers can be analyzed to ensure almost all of the EPID image gantry angles are within $\pm 1^{\circ }$ of the true gantry angle. This accuracy coincides with the AAPM‐recommended tolerance for gantry angle precision. Without the analyses proposed here, the EPID image header angles demonstrate an uncertainty as large as $\pm 3^{\circ }$. The methods discussed in this study can be applied to any EPID‐equipped linac. This study provides a critical step towards *in vivo* patient dose reconstruction during arc IMRT by ensuring the gantry angles associated with the EPID images are accurate and precise.

## ACKNOWLEDGMENTS

The authors would like to thank the Nuclear Electronics and Medical devices staff at CancerCare Manitoba for their assistance in completing this project and team leader of image acquisition at Varian Medical Systems, Daniel Morf. CancerCare Manitoba and the University of Manitoba's Faculty of Science and Graduate Studies funded this project.
